# Adherence to hemophilia patients with prophylaxis: Veritas-Pro and psychometric properties adapted to Turkish

**DOI:** 10.1371/journal.pone.0288625

**Published:** 2023-08-09

**Authors:** Zuhal Mehrekula Demirci, Nilufer Demiral Yilmaz, Hale Bulbul, Fatma Keklik Karadag, Fatos Dilan Atilla, Guray Saydam, Fahri Sahin

**Affiliations:** 1 Center of Hemophilia and Thrombosis, Ege University Hospital, Izmir, Turkey; 2 Department of Medical Education, Ege University School of Medicine, Izmir, Turkey; Mugla Sitki Kocman Universitesi, TURKEY

## Abstract

The Validated Hemophilia Regimen Treatment Adherence Scale—Prophylaxis (VERITAS-Pro) assesses adherence to prophylaxis treatment recommendations in hemophilia patients. This study aimed to adapt the VERITAS-Pro into Turkish and evaluate its reliability and validity. The research design used is a psychometric study. A convenience sample of 102 patients with hemophilia A or B was followed by the Aegean Adult Hemophilia and Thrombosis Center. The VERITAS-Pro was adapted to Turkish in six steps, including forward- and back-translation, committee review, and reliability and validity analysis. Based on the confirmatory factor analysis, modification indices suggested discrepancies amongst items, which were improved upon the removal of items 11 and 15. Findings from this alternative model are: χ^2^/df = 1.34; RMSEA = 0.05; SRMR = 0.09; and IFI = 0.92. The alternative model showed high adherence rates. Cronbach’s alpha value for the Turkish version was found to be 0.83. The test-retest reliability of the Turkish scale ranged from 0.31 to 0.78. All items discriminated significantly between participants who were more adherent and those who were less adherent (t = 23.53; p<0.01). Translation of the VERITAS-Pro into local languages enables more accurate measurement of treatment adherence among people with hemophilia and facilitates cross-cultural comparison studies. According to the validity and reliability evidence obtained, the psychometric properties of the Turkish version of the VERITAS-Pro are suitable.

## Introduction

Hemophilia is a lifelong bleeding disorder caused by a deficiency in a coagulation factor, specifically factor VIII (FVIII) or factor IX (FIX). It is marked by a tendency to bleed into joints, muscles, and mucosal membranes. The most debilitating complication of hemophilia is the development of a painful hemophilic arthropathy resulting from recurrent musculoskeletal bleeding events, which are often preventable with regular treatment. Hemophilia is managed episodically (when bleeding occurs) with plasma-derived or recombinant coagulation factor concentrates or prophylactically with factor concentrates or the monoclonal antibody emicizumab. International guidelines recommend prophylaxis for patients with severe hemophilia [1]. The main benefits of prophylaxis include reducing the number of bleeding events and preventing hemarthropathy [2–5]. However, adherence to prophylactic treatment regimens can be a challenge. Nonadherence to prophylactic treatment can cause an increase in the frequency, duration, and severity of bleeding events, but it also leads to negative socioeconomic outcomes [3, 6, 7]. Among patients with severe hemophilia using prophylaxis, reported adherence rates (calculated by a variety of methods) range from 44% to 96% [2–6]. Based on interviews with hemophilia patients, prophylaxis adherence is facilitated by acceptance of hemophilia, the experience and fear of symptoms, comprehension of hemophilia and prophylaxis, and possession of planning and infusion skills [8]. Schrijvers et al. (2013) identified the motivators of adherence to prophylaxis as the patient’s belief in the need for treatment, a positive relationship with a health care professional, and previous experience of bleeding [9].

Among the most common parameters used to evaluate adherence to prophylaxis therapy are joint bleeding frequency, joint examination, patient treatment logs, and pharmacy records [10]. However, these are unreliable measures, and nurses report difficulty using them to assess and monitor patient adherence [11]. In 2010, Duncan et al. published the Validated Hemophilia Regimen Treatment Adherence Scale-Prophylaxis (VERITAS-Pro), a measure of adherence to prophylaxis regimens among persons with hemophilia. VERITAS-Pro items were developed based on a survey of hemophilia care specialists and a patient focus group, and the scale was validated with patient treatment logs and global adherence ratings provided by patients and providers. The VERITAS-Pro is a valid and reliable measure of adherence to prophylactic treatment regimens in hemophilia [12]. It has been widely utilized in clinical practice and in research.

To enable accurate measurement of treatment adherence among persons with hemophilia in Turkey and to facilitate cross-cultural comparison studies, there is a need for a culturally and linguistically valid Turkish translation of the VERITAS-Pro. Cross-cultural and linguistic validation are processes to ensure the production of an appropriate translation of the original instrument that conveys the intended meaning of the original in a way that is culturally and linguistically familiar to the individual completing the instrument [13]. This study aimed to adapt the Validated Hemophilia Regimen Treatment Adherence Scale-Prophylaxis (VERITAS-Pro) into Turkish and evaluate its validity and reliability.

## Materials and methods

### Study design

The research is psychometric design. The study evaluated the psychometric properties of the Turkish version of the VERITAS- Pro. Using convenience sampling, 102 hemophilia A and B patients (88 hemophilia A and 14 hemophilia B) who were followed up at the Ege Adult Thrombosis Center and received prophylactic treatment for at least 2 years or longer were included in the study.

The protocol for this research has been approved by a suitably constituted Medical Research Ethics Committee of Ege University Faculty of Medicine, Approval No: 18–1.1/24, and it conforms to the provisions of the Declaration of Helsinki. Prior to inclusion in the study, all volunteers signed a written informed consent form. Written permission to adapt the VERITAS-Pro was obtained from the Indiana Hemophilia and Thrombosis Center via written communication with Natalie Duncan, MPH.

#### VERITAS-Pro (original scale)

Published by Duncan et al. in 2010, the Validated Hemophilia Regimen Treatment Adherence Scale-Prophylaxis (VERITAS-Pro) consists of 24 items on six 4-item subscales, each of which relates to a distinct adherence domain (Time, Dose, Plan, Remember, Skip, Communicate). VERITAS-Pro is completed using a five-point Likert scale (always-never) response format. The scale is scored such that lower scores denote higher levels of adherence. The total score ranges from 24 (most adherent) to 120 (least adherent) [12].

#### Translation procedure

The symmetrical method for translation was utilized. According to this approach, six consecutive steps are followed [14]. The first five steps establish linguistic validity while the sixth step examines validity, and reliability.

In Steps 1 through 4, the VERITAS-Pro was translated into Turkish using a forward-backward translation process and review from a committee (Table 1). In step five, the pre-final Turkish translation was administered to 10 patients with hemophilia representing varied ages, education levels, and socio-economic status. In this cognitive debriefing, these patients answered the pre-final Turkish translation and evaluated items in terms of meaningfulness, readability, understandability, sentence length, and clarity of meaning. After this evaluation, the conceptual and content equivalence of the items in the pre-final Turkish translation were discussed with the research team. A final Turkish translation was derived at the end of pilot testing and cognitive debriefing, and thus the Turkish version of the VERITAS-Pro was ready for application in the study sample.

**Table 1:** The process of the VERITAS-Pro translation into Turkish.

Step 1: Forward translation by two independent translators
VERITAS-Pro was translated from English into Turkish by two bicultural translators who were fluent in both English and Turkish languages to generate two translations of the scale (Turkish 1 and Turkish 2).
Step 2: Comparison of the two forward translations by a third independent translator
The items and response options of the two forward translations (Turkish 1 and Turkish 2) were compared against the original English VERITAS-Pro by a third independent translator. Upon achieving consensus (the items in terms of meaningfulness, readability, comprehensibleness, sentence length, and clarity of meaning) among the three translators from Steps 1 and 2 and the research team, the preliminary initial translation of the scale in Turkish (PI-Turkish) was generated.
Step 3: Blind backward translation of the preliminary initial translation
The PI-Turkish was translated back into English by two additional independent translators who had not seen the original version of the VERITAS-Pro. This generated two back-translations of the scale (B-Turkish 1 and B-Turkish 2).
Step 4: Comparison by committee of the two back-translations of the scale
The items and response format of the two back-translations (B-Turkish 1 and B-Turkish 2) were compared by a committee with the original English scale. The committee included one methodologist (NDY), seven hemophilia care specialists, and the four bilingual and bicultural translators involved in Steps 1 and 3. The committee evaluated the backward translations and developed the pre-final Turkish version of the scale for pilot and psychometric testing.

#### Reliability and validity testing

In the sixth step of the cultural and linguistic validation, the reliability and validity of the Turkish version of the VERITAS-Pro were assessed in a convenience sample. Data were collected between April and June 2019 from patients with hemophilia who consented to participate. In addition to the Turkish version of the VERITAS-Pro, patients also completed a cover sheet containing sociodemographic information. The forms were self-administered by patients and required approximately 15 minutes to complete.

Internal consistency reliability and test-retest reliability of the Turkish version of the VERITAS-Pro were evaluated. For test-retest reliability, the Turkish version of the VERITAS-Pro was completed a second time by 20 patients two weeks after the first completion. In addition, we compared the total scores of the lower (27% undermost) and upper (27% uppermost) groups to examine whether each Turkish version of the VERITAS-Pro item discriminated between both groups.

#### Data analysis

In the study, descriptive statistics, confirmatory factor analysis for construct validity, Cronbach’s alpha (α) for internal consistency reliability, and Pearson product-moment correlation coefficients (r) for test-retest reliability were calculated.

We performed confirmatory factor analysis (CFA) using structural equation modeling (SEM) to test the construct validity of the Turkish version of the VERITAS-Pro. As in the original scale, we tested the measurement model consisting of a six-factor structure. We used the following indexes of adherence to the model: chi-square/df (χ^2^/df), the root mean square error of approximation (RMSEA), the standardized root mean square residual (S-RMR), and the incremental fit index (IFI) [15].

We used item-total correlation and compared the scores of the undermost and uppermost performing groups by means of a t-test. All outcomes were considered statistically significant with a p-value of <0.05. Data analyses were performed with IBM SPSS Statistics, version 21 (IBM Armork, New York, USA), and LISREL, version 8.80.

## Results and discussion

Enrolled were 102 patients with hemophilia A or B. The characteristics of patients are listed in Table 2.

**Table 2:** Patient characteristics.

		n (%)
Diagnosis	Hemophilia A	88 (86.3)
	Hemophilia B	14 (13.7)
Age at diagnosis	<1 year	48 (47.1)
	1–5 years	29 (28.4)
	>5 years	25 (24.5)
Education	Elementary school	21 (20.5)
	High school	53(52)
	University	28 (27.5)
Factor preparation used	Recombinant	42 (41.2)
	Plasma-derived	60 (58.8)
Who administers the factor	Self-administered	68 (66.7)
	Family member	3 (2.9)
	Health care provider	14 (13.7)
	Other	17 (16.7)
Practices self-infusion	Yes	71 (69.6)
	No	31 (30.4)
Where the patient administered the drug	Home	74 (72.5)
	Workplace	3 (2.9)
	Hospital	12 (11.8)
	Home and workplace	13 (12.7)
Time of infusion	Starting the day	66 (64.7)
	When he or she rests	36 (35.3)
The number of bleeding episodes in the last six months	No bleeding	6 (5.9)
	1–2 bleedings	31 (30.4)
	3–5 bleedings	37 (36.3)
	More than 5 bleedings	28 (27.5)
Patient-reported difficulty preparing an infusion	He or she has difficulty	59 (57.8)
	Never had difficulty	43 (42.2)
Orthopedic intervention	Yes	33 (32.4)
	No	69 (67.6)

In the linguistic validation process, we identified a need to change only one clause. The term *“factor”* was added to facilitate understanding of the 15th item in the scale. Table 3 presents the recommended clause for Turkish translation.

**Table 3:** Recommended clause for Turkish translation.

Original item	Recommended clause for Turkish translation
15. I remember to infuse on the schedule prescribed by the treatment center.	15. I remember to infuse ***factor*** on the schedule prescribed by the treatment center.

**Fig 1:**
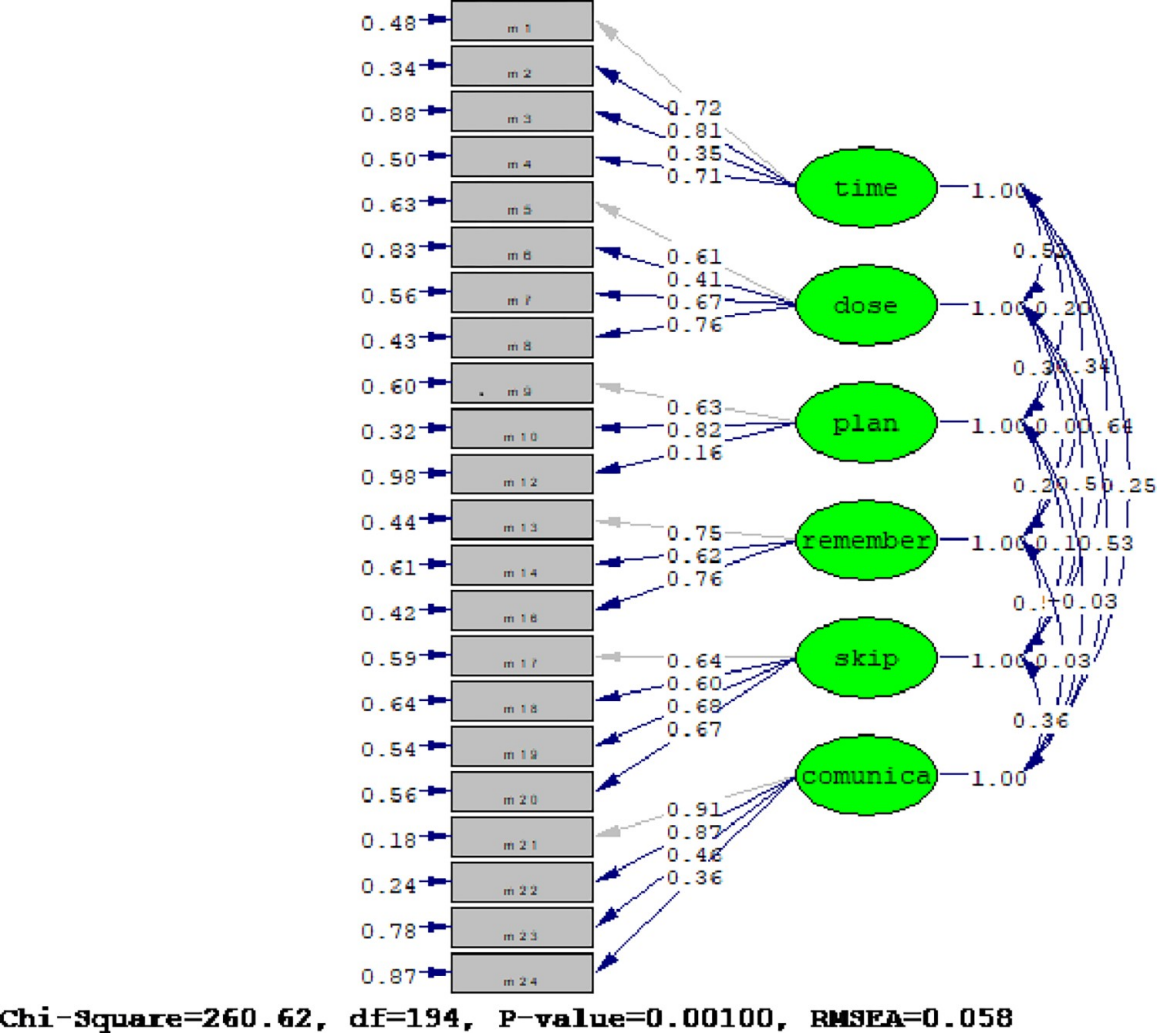
The measurement model of the Turkish version of the scale.

A hypothetical measurement model (measurement model-1) was created with the same 6-factor structure as the original scale. Based on CFA, the measurement model-1 did not provide model-data fit. We observed the modification indices and removed two items (11 and 15). The alternative model (measurement model-2) showed higher adherence rates than the masurement model-1. Table 4 presents the CFA results for the hierarchical models. Factor loads of measurement model ranged from 0.16 to 0.91 (Fig 1).

**Table 4:** Confirmatory factor analysis results for the hierarchical models.

Goodness of fit index	Good fit	Cut-off for good fit	Alternative model (6 factor 22 items)	Hypothetical model (6 factor 24 items)
χ2 (df)	-	-	260.62 (194)	292.62 (215)
p-value	< 0.05	< 0.05	0.00	0.00
χ2/df	< 3	< 5	1.34	1.36
RMSEA	0<RMSEA<0.05	0.05<RMSEA<0.10	0.05	0.06
S-RMR	0≤SRMR≤0.05	0.05≤S-RMR≤0.10	0.09	0.11
IFI	0.95≤IFI≤1	0.90≤IFI≤0.95	0.92	0.90

X^2^ (Chi-square), Df (degrees of freedom), RMSEA (root mean square error of approximation), S-RMR (standardized root mean square residual), IFI (incremental fit index)

Cronbach’s alpha value (α) denoting the internal consistency reliability of the Turkish scale, was 0.83. The correlation coefficients (r) calculated for test-retest reliability of the Turkish scale ranged from 0.31 to 0.78. Reliability analyses are presented in Table 5.

**Table 5:** Descriptive statistics, discriminant validity, and reliability analysis of the Turkish version of the scale.

Factors	n = 102			Skewness	Kurtosis		n = 20	
	Mean	SD	min-max			α	r	p
Time	6.7	2.4	4–13	0.34	0.11	0.70	0.39	>0.01
Dose	5.8	2.3	4–14	0.04	0.09	0.74	0.77*	<0.01
Plan	7.3	2.2	4–12	0.43	0.12	0.72	0.58*	<0.01
Remember	8.8	2.6	4–16	0.62	0.14	0.71	0.39	>0.01
Skip	7.2	2.5	4–17	0.58	0.11	0.73	0.32	>0.01
Communicate	8.1	3.9	4–20	0.77	0.13	0.74	0.78*	<0.01
VERITAS-Pro	44.1	10.4	26–70	0.97	0.16	0.83	0.31	>0.01

*Correlation is significant at the 0.01 level

All items discriminated significantly between highly and lowly performing patients (t_(52)_ = 23.53; p<0.01) (Table 6).

**Table 6:** Upper %27 and lower %27 groups statistics.

Groups	n	$\bar{x}$	SD	df	t	p
Upper 27%	27	67.12	8.14	52	t = 23.53	0.00
Lower 27%	27	29.43	3.87			

In this study, a multidisciplinary committee of qualified research staff, hemophilia experts, and translators adopted a symmetrical six-step approach to translate the Validated Hemophilia Regimen Treatment Adherence Scale-Prophylaxis (VERITAS-Pro) into Turkish and to demonstrate cross-culturally and linguistically the validity, internal consistency reliability, test-retest reliability, and construct validity of the translated measure.

The study sample consisted of 102 patients, distributed according to different characteristics. In the VERITAS-Pro Spanish language adaptation study by Cuesta-Barriuso et al. and the Brazilian version study by Ferreira et al., the sample sizes were 73 patients and 32 patients, respectively [22, 23].

The linguistic validation study identified the need to make one small edit in the Turkish version of the VERITAS-Pro to ensure that the translated version maintained the integrity of meaning from the original English questionnaire.

Using a hypothetical model of item distribution derived from the original VERITAS-Pro validation study by Duncan et al. (2010), the construct validity of the Turkish VERITAS-Pro was tested using CFA. The CFA analysis showed that the measurement model-1 data did not have an acceptable fit. According to the modification indexes, items 11 and 15 were removed, and CFA was repeated with alternative model. Using alternative model, the χ2 / sd rate is < 2, indicating that the model data fit is good [16]. The RMSEA (root mean square error of approximation) value, which provides estimates independent of the sample size, is < 0.05 and indicates a good fit [17]. The standardized RMR value < 0.10 indicates an acceptable fit [18]. IFI (incremental fit index), which is another index and makes very reliable estimates, is > 0.90, which shows acceptable fit [19, 20]. When the goodness of fit index is evaluated, the model data fit of the tested model is established. In the study by Wife (2018), EFA was performed to evaluate the construct validity of VERITAS-Pro. According to the findings of this research, a 7-factor structure was defined as a result of EFA [21]. The fact that there was a problem with item 11 in both studies suggests that the wording of this item may warrant attention. Item 11 states, “I run out of factor and supplies before I order more.” In step five, the term "factor" was added to item 15, which is on the Remember subscale. However, there was a problem with this item in the construct validity study. Item 15 is “I remember to infuse on the schedule prescribed by the treatment center”. The goodness of fit indices for the alternative model (removing items 11 and 15) are quite strong and well within the ranges considered “good” for a model fit. This strongly supports the CFA and the structure of almost all (excluding items 11 and 15) VERITAS subscales and items. Additional testing of the revised VERITAS-Pro is recommended using both versions (with and without items 15 and 11) before concluding that these items should be removed.

In reliability analysis, the Turkish version of the VERITAS-Pro Cronbach’s alpha (α) value was found to be 0.80. Given the short length of VERITAS-Pro subscales, alpha ≥0.8 will be considered to reflect excellent internal consistency. This finding of the study is similar to the original scale study, Spanish and Brazilian versions of the VERITAS-Pro study [12, 22, 23]. According to the test-retest reliability findings, a significant correlation was found between the two implementations of the scale adapted to Turkish. There is moderate correlation for the "Dose", "Plan", and "Communicate" subscales. There is a low correlation between the “Time”, “Remember”, and “Skip” subscales and Total VERITAS-Pro. Previous studies have found a higher correlation between the two implementations for test-retest reliability. This difference may be due to the longer time between the two implementations [22]. Similar findings are found in the “Skip” subscale of the study conducted in the Brazilian sample [23]. Between the two implementations, patients may experience behavioral changes regarding the items in the “Skip” sub-scale. Because when patients feel good, they may not adhere to treatment. If there is a problem in the skip subscale, it is expected to be seen in the time subscale.

All items discriminated significantly between low- and high-performing patients. The reliability study findings show that the scale is a reliable measurement tool that can be adapted to Turkish.

This indicates the discrimination of items in the Turkish version of VERITAS-Pro.

In the adaptation of the scale’s language, the term ’factor’ was added to enhance comprehension of item 15. No other cultural modifications were made to the items. The findings of the study regarding validity and reliability indicate that the Turkish version of the VERITAS-Pro gives valid and reliable scores and that hemophilia patients can be used to assess adherence to prophylaxis.

The strength of this study is that a confirmatory factor analysis was performed for the construct validity of VERITAS-Pro. In addition, the use of excessive validity and reliability evidence to evaluate the psychometric properties of the VERITAS-Pro strengthens this study. Another important point is that the patients in the study sample are distributed according to different characteristics. The study has some limitations. Goodness of fit indices for CFAs can be strongly affected by the sample size, particularly for sample sizes < 100. 102 patients for a CFA is a relatively small N (especially given a 24-item scale), so any results should be considered exploratory. A self-administered questionnaire technique was used in this study.

## Conclusion

It is known that the effectiveness of treatment in diseases and the positive progression of the disease process are directly related to the patients’ adherence to the regimen. Non-adherence with treatment is an obstacle to improving health but also harms the country’s economy [24]. Therefore, the World Health Organization recommends developing strategies that improve adherence to the regimen [25].

For hemophilia patients’ adherence to prophylactic regimens, it is important to know the rates of non-adherence, the conditions that cause it, and the attempts that can be made to prevent treatment non-adherence. VERITAS-Pro is not limited to substances that evaluate the use of prescribed drugs only in adherence with treatment. It also covers a wider range of substances that assess the patient’s adherence to the recommendations of health professionals in the behavioral dimension. It is important to translate VERITAS-Pro into different languages in order to be able to globally assess hemophilia patients’ adherence to treatment and conduct comparison studies. According to the validity and reliability evidence obtained in this study, the psychometric properties of VERITAS-Pro, which has been adapted to Turkish, can be said to be appropriate.

VERITAS-Pro, adapted to Turkish, which was obtained from the study, will contribute to the data collection and evaluation processes related to the adherence of hemophilia patients to prophylaxis.

## Supporting information

S1 File The Turkish version of Veritas-Pro. (PDF)
