# Supplementary material for: Adherence to hemophilia patients with prophylaxis: Veritas-Pro and psychometric properties adapted to Turkish
Source: PLoS One. 2023 Aug 9;18(8):e0288625. doi: 10.1371/journal.pone.0288625 (PMC10411756; doi:10.1371/journal.pone.0288625)
Supplement: S1 File — (PDF) [file pone.0288625.s001.pdf]

Erişkin Hemofili Hastalarında Tedaviye Uyumun Ölçümlenmesi: Çok Merkezli "Veritas-Pro" Anket Çalışması

Gönüllü No:…………………………………………….

VERİTAS-PRO

Hemofilinin yönetimi, kolay olmayan bir görevdir. Aşağıda Hemofilinin nasıl yönetildiği ve profilaksi tedavisi ile ilgili sorular bulunmaktadır. Doğru veya yanlış cevap bulunmamakla birlikte sadece son 3 (üç) ayda aşağıdakilerden her birini ne sıklıkta yaptığınıza dair bilgi edinmek istiyoruz. Sizin için en önemli nokta; her soruyu mümkün olduğunca dürüstçe cevaplamanızdır. Lütfen aşağıdaki ölçeğe göre her bir soruyu cevaplayınız.

Her zaman: daima, hep yaparım, zamanın %100'ü

Sıklıkla: çoğu zaman, en az %75 oranında

Bazen: aralıklı olarak, en az %50 oranında

Nadiren: çok az, %25 oranında

Hiçbir zaman: asla, hiç yapmam

Zamana uyum

1- Belirlenen günlerde profilaksi tedavisini uygularım.

- o Her zaman
- o Sıklıkla
- o Bazen
- o Nadiren
- o Hiçbir zaman

2- Haftalık önerilen tedavi dozunun tamamını uygularım.

- o Her zaman
- o Sıklıkla
- o Bazen
- o Nadiren
- o Hiçbir zaman

3- Önerildiği gibi profilaksi tedavisini sabah uygularım.

- o Her zaman
- o Sıklıkla
- o Bazen
- o Nadiren
- o Hiçbir zaman

4- Takip edildiğim merkezin belirlediği takvime göre tedavi uygularım.

- o Her zaman
- o Sıklıkla
- o Bazen
- o Nadiren
- o Hiçbir zaman

Doz uyumu

5- Doktorumun önerdiği dozu uygularım.

- **o** Her zaman
- o Sıklıkla
- o Bazen
- o Nadiren
- o Hiçbir zaman

6- Reçete edilenden daha az dozu uygularım.

- o Her zaman
- o Sıklıkla
- o Bazen
- o Nadiren
- o Hiçbir zaman

7- Takip edildiğim merkeze haber vermeden doz miktarını arttırır ya da azaltırım.

- o Her zaman
- o Sıklıkla
- o Bazen
- o Nadiren
- o Hiçbir zaman

8- Önerilen toplam dozu uygulamak için uygun sayıda faktör kutusunu kullanırım.
- o Her zaman
- o Sıklıkla
- o Bazen
- o Nadiren
- o Hiçbir zaman

Planlama

9- Planlı davranırım ve evde yeterli faktör bulundururum.
- o Her zaman
- o Sıklıkla
- o Bazen
- o Nadiren
- o Hiçbir zaman

10- Evde olan faktör ve malzeme miktarını yakından takip ederim.
- o Her zaman
- o Sıklıkla
- o Bazen
- o Nadiren
- o Hiçbir zaman

11- Yeni faktör ve malzeme almadan önce var olan faktör ve malzemelerin tamamını bitiririm.
- o Her zaman
- o Sıklıkla
- o Bazen
- o Nadiren
- o Hiçbir zaman

12- Evdeki faktör ve malzeme miktarını yakın takip edebilmek için bir sistemim vardır.
- o Her zaman
- o Sıklıkla
- o Bazen
- o Nadiren
- o Hiçbir zaman

Hatırlama

13- Profilaksi uygulamayı unuturum.
- o Her zaman
- o Sıklıkla
- o Bazen
- o Nadiren
- o Hiçbir zaman

14- Profilaksi uygulamayı hatırlamak zordur.
- **o** Her zaman
- o Sıklıkla
- o Bazen
- o Nadiren
- o Hiçbir zaman

15- Takip edildiğim merkezde reçete randevusu olduğunda faktör uygulamayı hatırlarım.
- o Her zaman
- o Sıklıkla
- o Bazen
- o Nadiren
- o Hiçbir zaman

16- Önerilen ilaç uygulamalarını unuttuğum için kaçırdığım olur.
- o Her zaman
- o Sıklıkla
- o Bazen
- o Nadiren
- o Hiçbir zaman

Atlama

17- Profilaksi uygulamasını atlarım.
- o Her zaman
- o Sıklıkla
- o Bazen
- o Nadiren
- o Hiçbir zaman

18- Reçete edilenden daha az sıklıkta faktör uygulamayı tercih ederim.
- o Her zaman
- o Sıklıkla
- o Bazen
- o Nadiren
- o Hiçbir zaman

19- Eğer ilaç uygulamak için uygun değilsem, o gün uygulamayı atlarım.
- o Her zaman
- o Sıklıkla
- o Bazen
- o Nadiren
- o Hiçbir zaman

20- Önerilen uygulamaları atladığım için kaçırdığım olur.
- o Her zaman
- o Sıklıkla
- o Bazen
- o Nadiren
- o Hiçbir zaman

İletişim

21- Hemofili ya da tedavi ile ilgili sorularım olursa takip edildiğim merkezi ararım.
- o Her zaman
- o Sıklıkla
- o Bazen
- o Nadiren
- o Hiçbir zaman

22- Hemofili ile ilgili sağlık sorunlarım ya da değişiklikler olduğunda takip edildiğim merkezi ararım.
- o Her zaman
- o Sıklıkla
- o Bazen
- o Nadiren
- o Hiçbir zaman

23- Takip edildiğim merkezi aramak yerine kendi kendime tedavi ile ilgili kararlar alırım.
- o Her zaman
- o Sıklıkla
- o Bazen
- o Nadiren
- o Hiçbir zaman

24- Tıbbi herhangi bir işlem olduğunda (örneğin, diş çekimi, kolonoskopi incelemeleri, acil servis başvurusu ya da hastanede yatmam gerektiğinde) takip edildiğim merkezi ararım.
- o Her zaman
- o Sıklıkla
- o Bazen
- o Nadiren
- o Hiçbir zaman
